# The Great American Recession and forgone healthcare: Do widened disparities between African-Americans and Whites remain?

**DOI:** 10.1371/journal.pone.0189676

**Published:** 2017-12-27

**Authors:** Jasmine L. Travers, Catherine C. Cohen, Andrew W. Dick, Patricia W. Stone

**Affiliations:** 1 NewCourtland Center for Transitions and Health, University of Pennsylvania School of Nursing, Philadelphia, PA, United States of America; 2 Rand Corporation, Santa Monica, CA, United States of America; 3 Rand Corporation, Boston, MA, United States of America; 4 Center for Health Policy, Columbia University School of Nursing, New York, NY, United States of America; Harvard Medical School, UNITED STATES

## Abstract

**Objective:**

During the Great Recession in America, African-Americans opted to forgo healthcare more than other racial/ethnic groups. It is not understood whether disparities in forgone care returned to pre-recession levels. Understanding healthcare utilization patterns is important for informing subsequent efforts to decrease healthcare disparities. Therefore, we examined changes in racial disparities in forgone care before, during, and after the Great Recession.

**Design:**

Data were pooled from the 2006–2013 National Health Interview Survey. Forgone medical, mental, and prescription care due to affordability were assessed among African-Americans and Whites. Time periods were classified as: pre-recession (May 2006-November 2007), early recession (December 2007-November 2008), late recession (December 2008-May 2010) and post-recession (June 2010-December 2013). Multivariable logistic regressions of race, interacted with time periods, were used to identify disparities in forgone care controlling for other demographics, health insurance coverage, and having a usual place for medical care across time periods. Adjusted Wald tests were performed to identify significant changes in disparities across time periods.

**Results:**

The sample consisted of 110,746 adults. African-Americans were more likely to forgo medical care during the post- recession compared to Whites (OR = 1.16, CI = 1.06, 1.26); changes in foregone medical care disparities were significant in that they increased in the post-recession period compared to the pre-recession (OR = 1.17, CI = 1.08, 1.28 and OR = 0.89, CI = 0.77, 1.04, respectively, adjusted Wald Test p-value < 0.01). No changes in disparities were seen in prescription and mental forgone care.

**Conclusion:**

A persistent increase in forgone medical care disparities existed among African-Americans compared to Whites post-Great Recession and may be a result of outstanding issues related to healthcare access, cost, and quality. While health insurance is an important component of access to care, it alone should not be expected to remove these disparities due to other financial constraints. Additional strategies are necessary to close remaining gaps in care widened by the Great Recession.

## Introduction

Eliminating health disparities has been a national priority and an ongoing pursuit [1]. The U.S. Department of Health and Human Services defines health disparities as “differences in health outcomes that are closely linked with social economic and environmental disadvantage” [2]. A critical resource to addressing health disparities has been through the acquisition of affordable health insurance coverage. Because of the positive association between wealth and utilization of healthcare services, however, it is possible that economic factors unrelated to health insurance coverage may prevent specific racial groups (i.e., African-Americans) from utilizing needed services [3].

## Background

The economic recession from December 2007 to June 2009, now known as “The Great Recession” [4], disrupted financial stability in the U.S. and imposed obstacles to achieving and maintaining adequate health insurance coverage for many Americans. Furthermore during this time, financial burdens, loss of health insurance, and other hardships such as home foreclosure and food insecurity, resulted in many Americans forgoing healthcare services (not obtain healthcare that they thought they needed because of costs) at a higher rate than before the recession [5]. Forgone care was not just seen in the unemployed. Many who remained employed experienced loss of health insurance coverage during this period and/or had an associated decrease in healthcare services utilization [6]. While the Great Recession impacted the healthcare utilization patterns of Americans from all backgrounds, prior studies indicate that this crisis took a greater toll on African-Americans than on Whites [7, 8]. Mortensen and colleagues found office-based physician visits to be lower for African-Americans compared to Whites during the Great Recession while use of prescription drugs were highest among Whites [8]. Burgard and colleagues found African-Americans more likely to forgo medical care because of costs compared to Whites during the Great Recession [7]. Reduced care seeking behaviors among racial minorities during the Great Recession may contribute to existing health disparities. Disparities in the type of care used can also lead to increased emergency department visits, increased costs, and facility overcrowding, placing burden on the entire healthcare system [9, 10].

As economic recovery from the Great Recession was characterized by persistently high unemployment [11], the forgone healthcare patterns thought to be a result of unemployment and strained finances that were previously documented may have continued long after the economy formally recovered. Findings presented in a 2015 Commonwealth Report further suggests newly implemented mandates for insurance coverage may not be sufficient to close racial gaps in forgone care [12]. Understanding healthcare utilization patterns throughout the Great Recession and prior to the full implementation of 2014 Healthcare Reform can inform modifications and efforts to decrease healthcare disparities. The purpose of this study is to examine changes in disparities between non-Hispanic African-Americans and Whites in forgone healthcare before, during and after the Great Recession.

## Methods

### Data set

We used eight years of National Health Interview Survey (NHIS) data, which is an annual cross-sectional survey of U.S. households conducted by the National Center for Health Statistics [13]. Through in-person interviews, NHIS records detailed health, demographic and socioeconomic information of non-institutionalized civilians. Interviews take place with 35,000 households containing approximately 87,500 individuals annually.^10^ NHIS’ cluster sampling and weighting methodology allows for generation of data that are nationally representative of the population [14]. Because the data were publicly available, in aggregate, and deidentified, the methods of this study did not warrant Institutional Review Board approval.

### Analysis

Pooled NHIS data collected in years 2006–2013 were analyzed [15]. Observations missing any of the variables of interest were dropped and the sample was limited to adults, aged 25–64 years old interviewed within the time period of interest (May 2006-December 2013). This age group was chosen because these individuals are the ages of working adults and have been found to be most likely to experience forgone care because of costs as opposed to those who have reached retirement age or still maintain insurance under their parents’ plan. The independent variable of interest, race, was categorized as White and African-American. Individuals also identifying as Hispanic were removed from the sample. Demographic variables included as controls were gender, age, and highest educational attainment. Age was categorized as 25–34, 35–44, 45–54 and 55–64 years of age and highest educational attainment categories were less than high school completion, high school graduate or General Educational Development test, some college or associate’s degree, and bachelor’s degree or more; both categorizations have been used in previous research [7]. Whether individuals were covered with health insurance and/or had a usual place for medical care was also included as a set of control variables in the analysis. Time periods were categorized to align with previous research, based on the National Bureau of Economic Research (NBER) classification of the Great Recession: pre-recession (May 2006-November 2007), early recession (December 2007-November 2008), late recession (December 2008-May 2010) and post-recession (June 2010-December 2013) [7, 16].

The three dependent variables of interest were forgone medical care, mental care, and prescription care. These variables were operationalized using the NHIS self-reported answers to questions: “during the past 12 months, was there any time when you needed any of the following, but didn't get it because you couldn't afford it?” This question was asked of respondents once for each type of care.

Using Pearson χ^2^, we described the sample by examining bivariate associations between race and the following variables: gender, age, educational attainment, health insurance coverage, usual place for medical care, and forgone care. We then developed a multivariable logistic regression model for each dependent variable of interest. In these models, the time period indicators were fully interacted allowing us to test whether the model estimates describing disparities in forgone care changed across time periods among African-Americans compared to Whites (reference group). The models controlled for gender, age, education attainment, health insurance coverage, and usual place for medical care. To understand if these disparities among African-Americans changed significantly across time periods, we performed adjusted Wald tests. All analyses were conducted using Stata 13.1 statistical software with p < 0.05 as the level of significance [17]. Regression results are reported using odds ratios and 95% confidence intervals. Appropriate use of NHIS adult sample weights was confirmed with a representative of the National Center for Health Statistics’ Division of Health Interview Statistics.

## Results

The sample size over the four time periods consisted of 110,746 NHIS adult participants. Whites accounted for 78% (n = 86,752) of the sampled population while African-Americans, accounted for 22% (n = 23,994). In the descriptive analyses, there was variation in the gender, age, and education composition of the sample by race (see Table 1). Whites were more likely to be older (> 44 years of age, p < 0.001), have insurance coverage, and maintain a usual place for medical care (p < 0.001).

**Table 1:** Description of sample by race.

		White^a^ % (n = 86,752)	African-American^a^ % (n = 23,994)	*P-value
Gender			< 0.001
Female	50.7	54.8	
Age in Years			< 0.001
25–34	22.5	28.0	
35–44	23.9	26.0	
45–54	28.7	27.0	
55–64	24.9	19.1	
Education			< 0.001
< High school	7.4	14.1	
High school/GED	25.9	30.0	
Some college or associate’s degree	31.0	33.9	
Bachelors or higher	35.7	22.1	
Health Insurance Coverage			< 0.001
Covered	86.0	77.5	
Usual Place for Medical Care			< 0.001
Yes	85.4	83.0	
More than 1	1.1	1.0	
Forgone Care			
Medical care	9.2	12.9	< 0.001
Mental care	3.2	3.3	< 0.001
Prescription care	9.6	14.2	< 0.001

Note: Total Sample size: 110,746; GED = General Education Development

^a^non-Hispanic

*All p-values < 0.05 were considered statistically significant.

Source: The National Health Interview Survey Sample May 2006-December 2013

Table 2 shows the results of our multivariable logistic regression analysis with fully interacted time-period indicators. In these analyses, African-Americans, were more likely to forgo medical and prescription care in the post-recession time period when compared to Whites (OR = 1.17, CI = 1.08,1.28 and OR = 1.19, CI = 1.10,1.30, respectively). On the other hand, African-Americans were less likely to forgo mental care compared to Whites in the post-recession period (OR = 0.68, CI = 0.59,0.79). The c-statistic which measures the predictive accuracy of a logistic regression model was greater than 0.70 for each of the three models indicating that the final models have good predictive ability in predicting our forgone care outcomes [18]. We present odds ratios for the other covariates in the full model in supplementary material (S1 Table).

**Table 2:** Likelihood of forgone care among African-Americans^a^ compared to Whites^a^, with fully interacted time periods of the Great Recession (May 2006-December 2013).

	Forgone Medical Care OR (95% CI)	Forgone Mental Care OR (95% CI)	Forgone Prescription Care OR (95% CI)
Pre-recession^b^	0.89 (0.77, 1.04)	0.78 (0.59, 1.04)	1.15 (0.99, 1.34)
Early Recession^b^	1.13 (0.94, 1.37)	0.85 (0.63, 1.14)	1.23 (1.00, 1.50)
Late Recession^b^	1.07 (0.94, 1.22)	0.83 (0.64, 1.08)	1.14 (1.00, 1.30)
Post-recession^b^	1.16 (1.06, 1.26)	0.68 (0.59, 0.79)	1.19 (1.09, 1.30)

*Note*: Total sample size: 110,746; OR = odds ratio; CI = confidence interval; Results from multivariable logistic regressions controlling for education, age, gender, health insurance coverage, and usual place for medical care. Only data for interacted time periods with race shown. All p-values <0.05 were considered statistically significant.

^a^Non-Hispanic

^b^Pre-recession (May 2006-November 2007), Early Recession (December 2007-November 2008), Late Recession (December 2008-May 2010) and Post-recession (June 2010-December 2013).

Source: National Health Interview Survey Sample

Table 3 provides p-values for the adjusted Wald tests that examines if these racial disparities in the likelihood of forgone care changed significantly across time periods. Between African-Americans and Whites, the disparity in forgone medical care increased from pre-recession to post-recession (p < 0.01). When performing a joint test of disparities across the four time periods for each forgone care among African-Americans relative to White, we rejected the null hypothesis that disparities were unchanged in forgone medical care (p = 0.03). No significant changes in disparities were seen in prescription and mental forgone care.

**Table 3:** Comparisons of racial disparities in forgone health care among different time periods (P- values for adjusted Wald tests of difference-in-difference).

	Forgone Medical Care	Forgone Mental Care			Forgone Prescription Care
African-American^a^ vs White^a^					
Pre-recession vs early recession^b^	0.05		0.71	0.65	
Pre-recession vs late recession^b^	0.07		0.77	0.89	
Pre-recession vs post-recession^b^	<0.01		0.38	0.71	
Joint differences across all time periods	0.03		0.39	0.90	

Note

^a^non-Hispanic

^b^Pre-recession (May 2006-November 2007), Early Recession (December 2007-November 2008), Late Recession (December 2008-May 2010) and Post-recession (June 2010-December 2013).

All p-values <0.05 were considered statistically significant.

Source: National Health Interview Survey Sample

## Discussion

This study is the first to determine whether changes in the racial disparities in forgone healthcare during the Great Recession persisted in the years following. Furthermore, unlike prior work, we additionally controlled for health insurance coverage allowing us to identify disparities unexplained by insurance status alone [7].

We found that African-Americans were disproportionately affected after the Great Recession. They experienced increased financial constraints compared to Whites in obtaining needed medical care after the recession ended compared to the pre-recession. The steadfast increase in forgone care disparities among African-Americans compared to Whites found in this study may be a result of outstanding issues related to healthcare access, cost, and quality.

Decreased access to care for African-Americans compared to Whites is a common finding in the literature [19–21]. Addressing access solely through health insurance coverage, which is primarily the case with the 2014 Healthcare Reform, however, may ignore other major components that play a role in one’s ability to prioritize and utilize healthcare services when affordability is in question. A recent review by Levesque and colleagues conceptualized important dimensions to accessing perceived needed care as the ability to seek, reach, pay, and engage with care services [22]. Andersen’s behavioral model of health services use additionally describes several characteristics that determine access including: predisposing characteristics (e.g., demographics, social, and beliefs), enabling characteristics (e.g., health policy, financing, and organization), and need characteristics (e.g., environmental need, population health indices, perceived need, and evaluated need) specific to the environment and/or individual [23]. These researchers describe how such factors, better known as social determinants of health, influence whether or not healthcare need remains unmet. For example, decreased access to care related to affordability may come as a result of one not being able to afford to take time off from work to attend a healthcare appointment.

Healthcare cost relative to ability to pay, is also a concern. Increased disparities in forgone medical care between African-Americans and Whites during the post-recession period compared to the pre-recession found in this study may be explained by a ballooned wealth gap [22]. In recent reports median wealth of Whites increased by 2.4% between the years 2010 and 2013 (post-recession period), but decreased for African-Americans by 33.7% during this same period [24]. Despite access to insurance coverage, such disparities in wealth may effect one’s ability to finance co-pays, co-insurance, and deductibles associated with care visits [23]. Unfortunately, required contributions vary across healthcare plans including the current exchanges and may not be affordable for all especially those who remain hard-hit during the Great Recession; using different 2012–2013 data, authors of a recent Commonwealth Fund Report found that African-Americans were more likely to not visit a doctor due to cost when compared to Whites [12]. Further restructuring of contribution requirements according to one’s ability to pay as well as decreasing the gap in wealth disparities is critical to decreasing barriers to care associated with cost.

Poor quality healthcare services may also affect African-Americans’ choice to forgo needed medical care when one must prioritize what they can afford. This racial group generally experiences increased dissatisfaction with care compared to Whites [19, 20]. When perceiving healthcare quality as less than optimal, it is likely that racial minorities and those who are socioeconomically disadvantaged may place competing priorities ahead of healthcare when needing to balance multiple costs. Making accommodations for patients of racial minority backgrounds (e.g., linguistic and cultural) and implementing quality improvement programs have been found to improve care quality, decrease health disparities, and increase one’s likelihood to obtain needed services [25].

Consistent with other reports, no changes in disparities were identified in mental care among African-Americans compared to Whites [26–28]. In fact, researchers have found that some racial minorities including African-Americans use mental healthcare services less than Whites [29]. Further, experts posit that racial minorities may not perceive mental healthcare as a necessity [26–28]. Understanding the rationale for forgone mental care in these minority populations is particularly important to our society as a whole as life experiences generally requiring mental healthcare support (e.g., homelessness, poverty, incarceration, unemployment, violence, and racism) affect racial minority groups (i.e., African-Americans) more than Whites [29–37]. A better understanding of the perceptions of mental health and care among high-risk groups may facilitate access to needed mental care not previously recognized [38].

## Limitations

This study has limitations. First, the data used are self-reported and subject to the recollection of the individuals interviewed. Despite this limitation, a study was conducted that compared data from the NHIS to data from the Behavioral Risk Factor Surveillance System and found the national estimates to be comparable [39]. Second, because this is cross-sectional data, associations could only be examined without temporality. We did however, examine multiple time periods and employed an interaction analysis which allowed us to identify significant changes in disparities over time. Third, these data do not contain information on reasons why cost influenced the decision to forgo care (e.g., lack of funds for a co-payment and/or transportation to obtain services or inability to afford time off from work to seek care). This is a limitation of the national data which warrants independent study of these questions. For example, a recent study conducted by Sommers et al. found that lack of available appointments were associated with cost-related delays in care [40].

## Conclusion

Disparities in the likelihood of forgone care have increased from pre-recession levels between African-Americans and Whites after the Great Recession. As higher rates of morbidity, disability, and mortality are pervasive among this racial group compared to all others, these findings have important policy implications [19]. Direct attention to the needs of African-Americans related to access, cost, and quality is necessary to combat ongoing disparities in medical care obtainment and ultimately health disparities. While health insurance is an important component of access to care, it alone should not be expected to remove increased disparities in forgone care due to financial constraints [12].

Policy initiatives that may address healthcare inequity issues beyond insurance coverage include: increasing workforce diversity; ensuring cultural and linguistic competence among healthcare workers; providing community outreach; promoting community participation; and implementing cost sharing insurance plans [41]. Future research should assess the specific financial burdens (i.e., transportation, co-payments, and opportunity cost of lost wages during care visits) along with predisposing, enabling, and need characteristics that augment increased disparities in forgone care among African-Americans.

## Supporting information

S1 Table (XLSX)
